# Synthesis and Characterization of Reduced Graphene Oxide-Supported Nanoscale Zero-Valent Iron (nZVI/rGO) Composites Used for Pb(II) Removal

**DOI:** 10.3390/ma9080687

**Published:** 2016-08-12

**Authors:** Mingyi Fan, Tongjun Li, Jiwei Hu, Rensheng Cao, Qing Wu, Xionghui Wei, Lingyun Li, Xuedan Shi, Wenqian Ruan

**Affiliations:** 1 Guizhou Provincial Key Laboratory for Information Systems of Mountainous Areas and Protection of Ecological Environment, Guizhou Normal University, Guiyang 550001, China; fanmingyifmy@163.com (M.F.); tongjunxiaoyu@163.com (T.L.); 18230825324@163.com (R.C.); wq0851@126.com (Q.W.); lingyunli1989@126.com (L.L.); xuedanshi1991@163.com (X.S.); wenqianruan@yahoo.com (W.R.); 2 Department of Applied Chemistry, College of Chemistry and Molecular Engineering, Peking University, Beijing 100871, China; xhwei@pku.edu.cn

**Keywords:** Pb(II) ions, nanoscale zero-valent iron, graphene, removal, response surface methodology

## Abstract

Reduced graphene oxide-supported nanoscale zero-valent iron (nZVI/rGO) composites were prepared by chemical deposition method and were characterized by scanning electron microscopy (SEM), X-ray diffraction (XRD), Raman spectroscopy, N_2_-sorption and X-ray photoelectron spectroscopy (XPS). Operating parameters for the removal process of Pb(II) ions, such as temperature (20–40 °C), pH (3–5), initial concentration (400–600 mg/L) and contact time (20–60 min), were optimized using a quadratic model. The coefficient of determination (*R*^2^ > 0.99) obtained for the mathematical model indicates a high correlation between the experimental and predicted values. The optimal temperature, pH, initial concentration and contact time for Pb(II) ions removal in the present experiment were 21.30 °C, 5.00, 400.00 mg/L and 60.00 min, respectively. In addition, the Pb(II) removal by nZVI/rGO composites was quantitatively evaluated by using adsorption isotherms, such as Langmuir and Freundlich isotherm models, of which Langmuir isotherm gave a better correlation, and the calculated maximum adsorption capacity was 910 mg/g. The removal process of Pb(II) ions could be completed within 50 min, which was well described by the pseudo-second order kinetic model. Therefore, the nZVI/rGO composites are suitable as efficient materials for the advanced treatment of Pb(II)-containing wastewater.

## 1. Introduction

Heavy metals are one of the most important classes of pollutants due to their wide sources, non-degradability and high toxicity [1,2]. With the development of industrialization and urbanization, the needs for metals have increased drastically, and therefore have created a widespread contamination of heavy metals to water bodies and soils, especially in developing countries [3]. Lead is one of the most dangerous heavy metals, and is used as an industrial raw material for storage battery manufacturing, printing, pigments, fuels, photography materials and explosives manufacturing [4]. The use of lead compounds also causes large amounts of industrial wastewater containing the high quantity of Pb(II) ions. Lead is a potent neurotoxin, therefore childhood lead poisoning has an impact on many developmental and biological processes, most notably intelligence, behavior, and overall life achievement [5]. The reason for this is that children can absorb 40% to 50% of an oral dose of water-soluble lead compared with 3% to 10% for adults. From 2009 to 2011, 10 major lead poisoning episodes caused by heavily polluted enterprises occurred in China, affecting more than 4000 children [6]. From 2015 to 2016, a public health crisis has resulted from the switching of the water supply from Lake Huron to a more corrosive water source from the Flint River, which has become an important environmental issue because of the Pb(II) pollution [7]. It was reported that the elevated blood lead levels (≥5 µg/dL) increase from 2.4% to 4.9% after the water source change, and neighborhoods with the highest water lead levels experienced a 6.6% increase in this region [5].

Because Pb(II) ions are non-biodegradable and tend to bioaccumulate in cells of living organisms, stricter environmental requirements and urgent treatment methods are needed for their removal from water and wastewater [8]. Since primary treatment (grid, grit chamber and primary settling tank) and secondary treatment (biological treatment) cannot completely treat wastewater, tertiary treatment (advanced treatment) of Pb(II) ions in wastewater was carried out by several methods, such as nanofiltration (NF), reverse osmosis (RO), solvent extraction, reduction, ion-exchange, electro-chemical reduction, evaporation, precipitation and adsorption. Among these existing methods, adsorption has become a popular and effective technique due to its better performance, availability of various adsorbents, ease of handling and no production of toxic secondary compounds [9]. One of major industrial-scale techniques for the removal of Pb(II) ions is the chelating resin as a traditional adsorption tool, which has been used for the recovery of Pb(II) ions in wastewater for many years. Its economic cost is still relatively high when the metals are dissolved in the large volume of solution with a relatively low concentration [10]. Furthermore, traditional separation methods, including centrifugation and filtration, are time consuming and tedious. By addressing these problems, using high removal capacity and easy separation materials is an ideal approach to remove Pb(II) ions [11,12,13,14]. Nanomaterials with high efficiencies in the advanced treatment of wastewater can overcome the shortcomings of the traditional methods, which can replace or operate together with the traditional methods for advanced treatment of wastewater.

Nanoscale zero-valent iron (nZVI) is known to be a promising, effective reductant because of its large specific surface area, small particle size, excellent reactivity and high injectability into an aqueous solution. Despite being highly effective for heavy metals, bare iron nanoparticles tend to rapidly agglomerate and are highly susceptible to oxidation [15]. Bare iron nanoparticles lack durability and their mechanical strength is also weak. To overcome these shortcomings, technologies have been developed using some materials as mechanical supports to enhance the dispersion of nZVI particles [16]. Many studies have been carried out on the use of porous materials to support nZVI, for example, nZVI particles supported on resin, were used to remove Pb(II) ions in aqueous solution, and the results showed that the reaction rates for Pb(II) ions were up to 30 times higher than that using iron fillings or powder [17]. Kaolinite was selected as the supporting material loaded with 20 wt % nZVI (K-nZVI), and the removal efficiency of Pb(II) ions was obviously higher than using bare nZVI [18]. The reduced graphene oxide-supported nanoscale zero-valent iron (nZVI/rGO) composites have been considered simple and effective for the treatment of contaminants, such as cadmium, uranium, arsenic and trichloroethylene [19,20,21,22,23,24]. The nZVI/rGO composites were also used to remove Pb(II) ions in aqueous solution, and the operating parameters, such as contact time, solution pH, initial Pb(II) concentration and temperature, have been investigated [25,26]. However, there is no information available in the literature regarding the optimization of Pb(II) ions removal by nZVI/rGO composites.

To achieve an optimal control and management of Pb pollution, new concepts involving efficient operation and design should be developed and understood [27]. Hence, a number of tools, such as artificial neural network (ANN), response surface methodology (RSM), partial least square (PLS), modified simplex method (MSM) and uniform experimental design (UED), can be selected to optimize the process for the removal of Pb ions. The past decade has especially seen a host of data analysis tools based on biological phenomena developed into well-established modeling techniques, such as artificial intelligence (AI) and evolutionary computing [28]. ANN is one of the major tools for AI that has been employed to predict the solid–liquid interface adsorption capacity of activated carbon for the removal of phenol from aqueous solution [29]. However, this study required a large number of batch experiments to be performed. The experimental design technique is a powerful tool in the field of engineering, which helps in better understanding and improving efficiency of the process [9]. RSM is a set of mathematical and statistical techniques that has been found to be a useful method for studying the effect of several factors influencing the response as well as optimizing the parameters in the removal process. RSM also identifies the relationship between the controllable input parameters and the response variable. Compared with the orthogonal experiment, the advantage of the RSM is that it can continuously analyze the various levels of the experiment, while the orthogonal experiment can only analyze the experiment point. Another important advantage for the use of statistical models in process optimization is that it requires very less number of experiments to be performed and hence, minimizes time, as well as cost [30]. Box–Behnken design is a spherical, revolving RSM design that consists of a central point and the middle points of the edges of the cube circumscribed on the sphere [31]. It consists of three interlocking 2^2^ factorial designs having points, all lying on the surface of a sphere surrounding the center of the design [32]. RSM has also been applied for the optimization of several chemical and physical processes [33,34], and the number of experiments is decided accordingly.

The objectives of the present study were: (a) to prepare and characterize the nZVI and nZVI/rGO; (b) to optimize the process parameters (temperature, pH, initial concentration and contact time) for maximum removal efficiency using the Box–Behnken design; and (c) to fit the obtained removal data to the adsorption isotherms and kinetic models. In addition, the mechanisms for the removal of Pb(II) ions by the nZVI/rGO composites were investigated based on the X-ray photoelectron spectroscopy (XPS) analysis.

## 2. Materials and Methods

### 2.1. Materials and Instruments

All chemical reagents (NaBH_4_, H_2_SO_4_, HCl, NaOH and FeSO_4_·7H_2_O) used in this study were of AR grade. Pb(NO_3_)_2_·4H_2_O (Mw = 331.21 g/mol) was dissolved in deionized water to prepare the stock solution for Pb(II) ions, which was further diluted with deionized water to the required initial concentrations for practical use. Graphite powder (<30 μm) was supplied by Sinopharm Chemical Reagent. Flame atomic absorption spectrophotometer (WFX-210) used in this study was made by Ray Leigh Corporation, Beijing, China.

### 2.2. Preparation of nZVI and nZVI/rGO

The synthesis of graphene oxide (GO) was achieved via the chemical exfoliation of graphite, using the improved Hummer’s method [26,35,36]. The nZVI/rGO composites were prepared via the reduction of FeSO_4_·6H_2_O and graphene oxide with NaBH_4_ (mass ratio, carbon:iron = 1:2). Graphene oxide (1 g) was dispersed in 300 mL deionized water by 2 h ultrasonication bath and 10 g FeSO_4_·6H_2_O was added into the above solution, then the mixture was continuously stirred by magnetic stirrer for 12 h. NaBH_4_ solution (5.46 g/50 mL) was dropwise added into the solution at 25 °C, which was continuously stirred for 30 min. The prepared materials were separated from the liquid phase via centrifugation and the resulting black solid was vacuum-dried at 50 °C overnight. The nZVI was prepared similarly to the nZVI/rGO, without the addition of GO.

### 2.3. Characterization

Scanning electron microscope (SEM) images of nZVI and nZVI/rGO composites were taken using a NoVa™ Nano SEM 250 (FEI, Hillsboro, OR, USA). X-ray diffraction (XRD) patterns of nZVI/rGO composites and nZVI were measured on a Philips Analytical X-ray (Lelyweg 1 7602 EA Almelo) with a Cu-Kα X-ray source (generator tension = 40 kV, current 40 mA). Continuous scans from 5° to 90° of 2θ were collected at a scan rate about 5° of 2θ/min. Raman spectra of nZVI and nZVI/rGO composites were recorded at 532 nm by the Renishaw inVia with semiconductor laser. The Brunauer–Emmett–Teller (BET) surface areas of nZVI and nZVI/rGO composites were obtained from N_2_ adsorption isotherms at 77 K with a Micromeritics 3 Flex surface characterization analyzer. The nZVI and nZVI/rGO composites were characterized by X-ray photoelectron spectroscopy (XPS) using a hemispherical energy analyzer Phoibos 100/150 (SPECS, Berlin, Germany) (Al Kα X-ray source, 1486.6 eV). The XPS spectra peak energies were corrected with the C 1s peak at 284.8 eV as a reference.

### 2.4. Batch Experimental Program

Batch experiments were performed for the removal of Pb(II) ions by the nZVI/rGO composites in 100 mL centrifugal tubes, in which 0.03 g of nZVI/rGO composites were added into 50 mL of Pb(II) solution with the known pH and initial concentration (*C*_0_). The above solutions were subsequently shaken on temperature controlled water bath shaker at 200 rpm for desired temperature and contact time. According to the previous study [26], the NaNO_3_ solution was added to adjust the ionic strength (0.05 mol/L) to optimize the removal of Pb(II) ions. The pH value of the sample solution was adjusted by the addition of 0.1 mol/L HCl or 0.1 mol/L NaOH to conduct the batch experiments in desired pH.

The nZVI/rGO composites were separated from the solutions for all the sample, and the Pb(II) ions concentration in the solutions was analyzed by atomic absorption spectrophotometer (AAS). Blank experiment (50 mL of Pb(II) solution) was conducted simultaneously in similar conditions without adding the nZVI/rGO composites to measure Pb(II) ions adsorbed by a centrifugal tube. All experiments were carried out in triplicate to minimize any experimental errors, and then the average values of the replicate measurements were used in all analyses. The removal percentage and the removal capacity of Pb(II) ions were calculated using Equations (1) and (2).

(1) $$Y=(C_{0}-C_{t})/C_{0}\times 100$$

where *Y* is the removal percentage of Pb(II) ions, *C*_0_ is the initial concentration of Pb(II) ions (mg/L), and *C_t_* is the concentration of Pb(II) ions after removal.

(2) $$q_{e}=(C_{0}-C_{t})\times V/m_{s}$$

where *q_e_* is the removal capacity of Pb(II) ions (mg/g), *V* is the volume of the solution used, and *m_s_* is the weight of nZVI/rGO composites (g).

### 2.5. Box–Behnken Design

In the present work, the 3-level 4-factor Box–Behnken experimental design was applied to research and validate process parameters influencing the removal of Pb(II) ions by nZVI/rGO composites. Namely, temperature (*X*_1_), pH (*X*_2_), initial concentration (*X*_3_) and contact time (*X*_4_) were selected as independent variables, and the removal percentage of Pb(II) ions (*Y*) was considered as the dependent variable. The factor levels were coded as −1 (low), 0 (central point) and 1 (high). The number of experiments needed to investigate the optimization of removal process are 81((3)^4^), which was reduced to 29 by using a Box–Behnken experimental design. The results were analyzed using the coefficient of determination (*R*^2^), Pareto analysis of variance (ANOVA) and statistical and response plots [32]. The method of fitting is used for the response surface modeling in order to obtain the equation. The most commonly used fitting method is the polynomial method, including linear polynomial, quadratic polynomial, cubic polynomial and high number of polynomials. The relation of simple factors can be described by linear polynomial, while the interaction of multiple factors can be expressed by quadratic polynomial and more complex interactions of multiple factors can be represent by cubic polynomial or high number of polynomials. Among these methods, quadratic polynomial is commonly used for response surface modeling. Based on the obtained polynomial, the response surface plot and contour for each of the response surfaces at different rake angles can be drawn to achieve the extreme value. A non-linear regression method was used to fit the experimental results to the second order polynomial and to identify the relevant model terms. Therefore, considering all the linear terms, square terms and linear by linear interaction items, the experimental data were analyzed by the response surface regression (RSREG) procedure to fit the following second order polynomial model:

(3) $$Y=a_{0}+a_{1}X_{1}+a_{2}X_{2}+a_{3}X_{3}+a_{4}X_{4}+a_{12}X_{1}X_{2}+a_{13}X_{1}X_{3}+a_{14}X_{1}X_{4}+a_{23}X_{2}X_{3}+a_{24}X_{2}X_{4}+a_{34}X_{3}X_{4}+a_{11}{X_{1}}^{1}+a_{22}{X_{2}}^{2}+a_{33}{X_{3}}^{2}+a_{44}{X_{4}}^{2}$$

where *Y* represents the removal percentage of Pb(II) ions; *X*_1_, *X*_2_, *X*_3_, and *X*_4_ represent independent variables; *a*_0_ is a constant offset term; and *a*_1_, *a*_2_, *a*_3_ and *a*_4_ are linear coefficients. However, *a*_12_, *a*_13_, *a*_14_, *a*_23_, *a*_24_, and *a*_34_ are interaction coefficients, and *a*_11_, *a*_22_, *a*_33_ and *a*_44_ represent the quadratic coefficients, which are computed from the predicted responses [37]. All experimental ranges and levels of independent variables chosen are collected in Table 1, which are based on the single factor experiments (Figure S1). The single factor experiments were carried out to provide a reasonable range for the independent variables of response surface experiments.

## 3. Results and Discussion

### 3.1. Characterization of nZVI and nZVI/rGO

**Figure 1:**
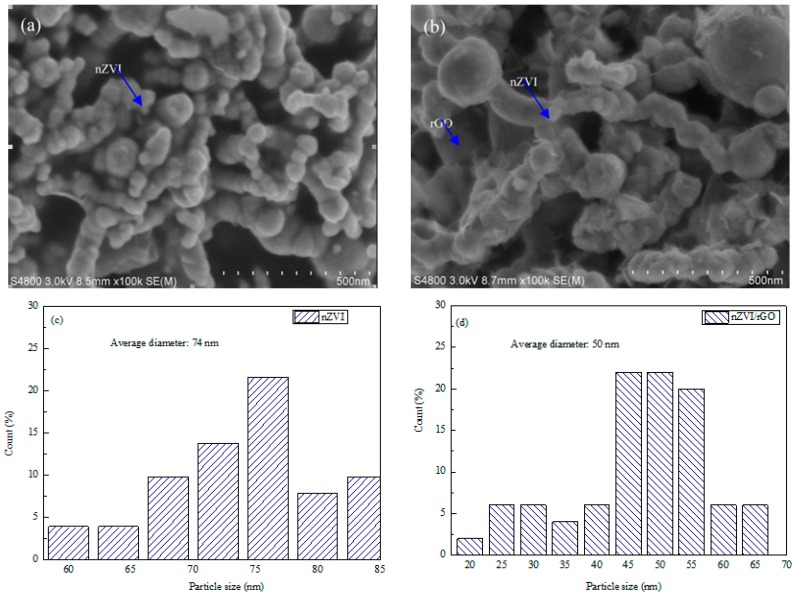
SEM images of nZVI (**a**) and nZVI/rGO composites (**b**); and size distributions calculated from SEM images of nZVI (**c**) and nZVI/rGO composites (**d**).

The SEM images show the morphology and nanoparticle distribution of nZVI and nZVI/rGO composites (Figure 1). It was found that the nZVI nanoparticles were aggregated (Figure 1a), while these nanoparticles were dispersed well on reduced graphene oxide (Figure 1b), because the rGO could enhance the dispersion of nZVI nanoparticles. The diameter distributions of nZVI and nZVI/rGO composites are concentrated in the range of 60–85 nm and 20–65 nm, and the average diameter of nZVI and nZVI/rGO composites are 74 nm and 50 nm (Figure 1c,d). This also indicated that the rGO can effectively prevent the aggregation of nZVI nanoparticles. The more SEM images of nZVI and nZVI/rGO composites are shown in Supplementary Materials (Figures S2 and S3).

**Figure 2:**
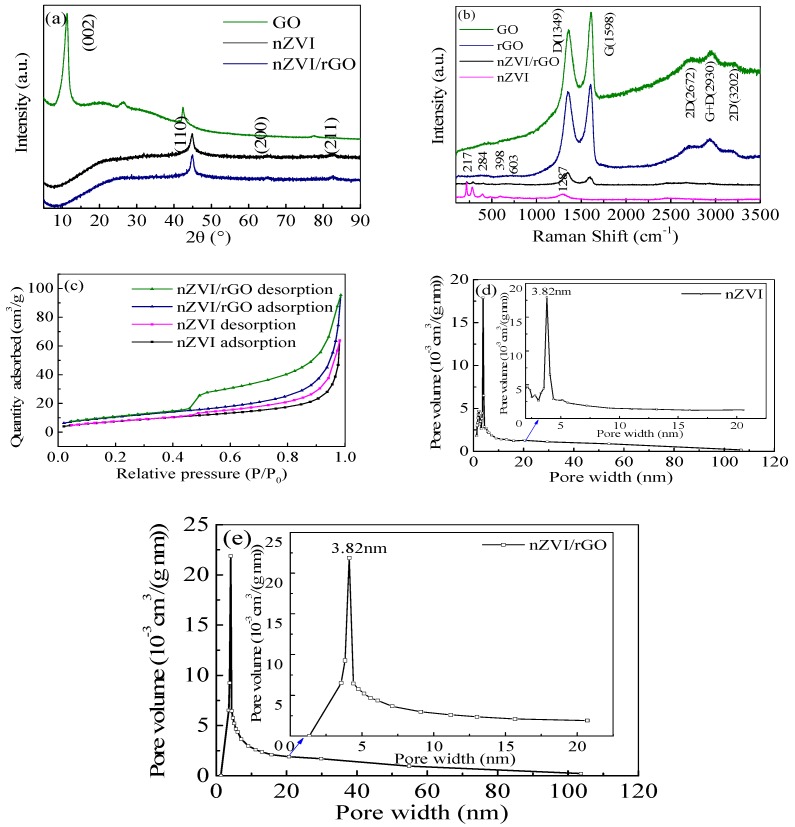
XRD patterns of nZVI and nZVI/rGO composites (**a**); Raman spectra of graphene oxide, reduction graphene oxide, nZVI and nZVI/rGO composites (**b**); the adsorption/desorption isotherms of the nZVI and nZVI/rGO composites (**c**); and BJH pore-size distribution curves of nZVI (**d**) and nZVI/rGO composites (**e**).

The X-ray diffraction patterns of GO, nZVI and nZVI/rGO composites are shown in Figure 2a. The GO pattern shows that the peak at 11° is attributed to the (002) crystalline plane of GO and the corresponding interlayer spacing is about 0.80 nm as a result of introduction of oxygenated functional groups on the carbon sheets. This peak became invisible after the reduction reaction in the nZVI/rGO composites, indicating the full reduction of GO. Moreover, this also confirmed that the nZVI was successfully supported by the rGO. The XRD patterns for the nZVI and nZVI/rGO composites reveal small size nanoparticles in the composites indicated by the peaks for α-Fe^0^ at the 2θ angles of 44.7° (110), 65.0° (200), and 82.6° (211) [38]. These peaks were consistent with the standard XRD data for the body-centered cubic (BCC) Fe (0) (JCPDS No. 010-87-0722). The iron oxides phase was not observed in the XRD pattern, because the crystallinity of iron oxides is low.

The Raman spectra of rGO, nZVI/rGO, GO, and nZVI are presented in Figure 2b. The rGO exhibited the two well known G and D peaks centered at 1348 cm^−1^ and 1598 cm^−1^, respectively. The Raman spectrum of nZVI/rGO composites was similar to that of rGO with sharp G and D peaks at 1346 cm^−1^ and 1595 cm^−1^, respectively. The intensity ratio of G to D peaks (*r* = *I*G/*I*D) is a measure of the disorder in the graphene. Disorder increases with the decrease of the ratio of *I*G/*I*D. The *I*G/*I*D ratio of GO, rGO and nZVI/rGO were 1.10, 1.06 and 0.86, which indicated that the interaction between nZVI and rGO induced a decrease of the order degree of carbon atoms in rGO. The decrease of the G peak corresponding to the graphite domain in rGO also provided a further evidence for the interaction in the nZVI/rGO composites. The 2D, D+G and 2D’ peaks were observed at 2672 cm^−1^, 2930 cm^−1^ and 3202 cm^−1^, which might indicate the layers of GO and rGO. The G and D peaks correspond to the integrity and defect level of graphene. The Raman spectra of the nZVI exhibited Raman peaks at 217 cm^−1^, 284 cm^−1^, 398 cm^−1^, 603 cm^−1^ and 1287 cm^−1^, which were attributable to Fe_2_O_3_. In the Raman spectra of nZVI/rGO composites, the peaks of Fe_2_O_3_ disappeared, which also indicated that nZVI was successfully supported by rGO.

Figure 2c shows the adsorption–desorption isotherms of the nZVI and nZVI/rGO composites. The isotherms of nZVI and nZVI/rGO composites exhibited a type of IV curve showing the presence of mesopore, and have a hysteresis loop at relative pressure between 0.42 and 0.98, indicating the pore size distribution in the mesoporous region. The BET specific surface areas of the nZVI and nZVI/rGO composites were 29 m^2^/g and 39 m^2^/g, respectively. The specific surface area of the nZVI/rGO composites was higher than that of the nZVI, which also indicated that rGO successfully prevented the aggregation of nZVI. The pore size distribution curves show that nZVI and nZVI/rGO composites possess one kind of mesopores with the size centered at 3.82 nm (Figure 2d,e).

**Figure 3:**
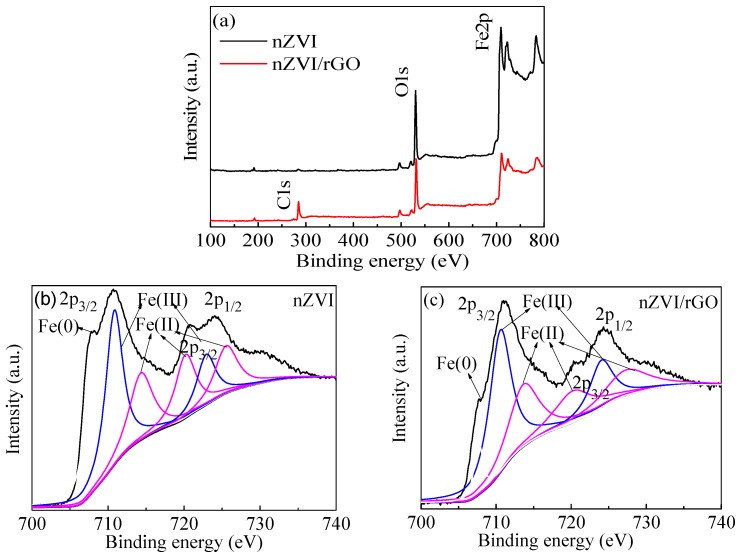
XPS analyses of the nZVI and nZVI/rGO composites: wide scan (**a**); high resolution spectra of Fe 2p in lab made nZVI (**b**); and high resolution spectra of Fe 2p in lab made nZVI/rGO (**c**).

The XPS spectra of both wide scan and high-resolution of Fe 2p for the nZVI/rGO composites and nZVI are shown in Figure 3. The XPS results of wide scan shown in Figure 3a illustrated that C, O and Fe were the major elements in the nZVI/rGO composites. The presence of C 1s peak also demonstrated that nZVI was supported by rGO. In the high-resolution spectra of Fe 2p (Figure 3b,c), the three peaks at approximately 711.20 eV, 720.60 eV and 724.80 eV were observed in both the nZVI/rGO composites and nZVI nanoparticles. These represent the binding energies of the Fe (2p_3/2_) and shake-up satellite (2p_3/2_ and 2p_1/2_) peaks, which indicated the existence of Fe(II) and Fe(III). The coexistence of a large fraction of iron oxides and a relatively small amount of nZVI confirms the core–shell structure of nZVI. Figure 3b,c also shows a small peak at 706.99 eV suggesting the 2p_3/2_ peak of zero valent iron (Fe^0^), since the surface was largely made up of iron oxides. The zero-valent iron peak at 706.99 eV was small in the XPS spectrum, probably because XPS is a surface analysis technique with only 2–5 nm probing depth.

### 3.2. Box–Behnken Analysis

RSM is an empirical modeling technique used to evaluate the relationship between the experimental and the predicted results [39]. In the present study, the Box–Behnken design was used to obtain a proper model for the optimization of the removal efficiency with four process variables (temperature, pH, initial concentration and contact time) at three levels. All experiments were conducted in triplicate to verify the precision for the removal of Pb(II) ions by nZVI/rGO composites [40]. Table 2 shows the value of the dependent and independent variables as well as the actual and predicted data for removal efficiency of every experiment, and a good agreement existed between the predicted results and those obtained from the experiments. The center point (0, 0, 0, 0) was repeated five times and similar results were obtained, indicating the satisfactory reproducibility of the data.

The response function with determined coefficients for the removal of Pb(II) ions is given below:

*Y*= −32.87 + 6.56*X*_1_ + 7.95*X*_2_ − 0.10*X*_3_ + 1.69*X*_4_ − 0.14*X*_1_*X*_2_ − 0.0003*X*_1_*X*_3_ − 0.05*X*_1_*X*_4_ + 0.004*X*_2_*X*_3_ + 0.10*X*_2_*X*_4_ + 0.0001*X*_3_*X*_4_ − 0.04*X*_1_^2^ − 1.08*X*_2_^2^ + 0.0001*X*_3_^2^ − 0.005*X*_4_^2^ (4)

### 3.3. Fitting of Second Order Polynomial Equations and Statistical Analysis

In order to evaluate the significance of the regression model, factors and their interactions, the analysis of variance (ANOVA) was performed. ANOVA is a statistical technique that subdivides the total variation in a set of data into component parts associated with specific sources of variation for the purpose of testing hypotheses on the parameters of the model [30]. The *F*-value is the ratio of the regression mean square and the real error mean square, which indicates the influence of each controlled factor on tested model [41,42]. Since the *p*-values of all the coefficients are *p* < 0.05, it is indicated that these coefficients are significant. The ANOVA for response surface quadratic model (Table 3) gives a *F*-value of 209.446, which implies that the terms have a significant effect on the response in the model. There is only a 0.01% chance that a large “Model *F*-value” could occur due to the noise. The lack of fit (LOF) is the variation of the data around the fitted model. The *F*-value for LOF is 5.085, which shows that the model used to fit response variables is significant and adequate to represent the relationship between the response and the independent variables. In this model, effects of the temperature (*X*_1_), pH (*X*_2_), initial concentration (*X*_3_) and contact time (*X*_4_) are significant (*p* < 0.05). Among these factors, the *F*-value of the contact time is 737.2203, which demonstrates that the removal percentage of Pb(II) is more related to the contact time in this model. Similarly, interactions are significant between the temperature and pH (*X*_1_*X*_2_), the temperature and initial concentration (*X*_1_*X*_3_), the temperature and contact time (*X*_1_*X*_4_), and the pH and contact time (*X*_2_*X*_4_). The effects of the temperature (*X*_1_^2^), pH (*X*_2_^2^), initial concentration (*X*_3_^2^) and contact time (*X*_4_^2^) are also significant (*p* < 0.05). The model also gives the coefficient of determination (*R*^2^) value of 0.9952, the adjusted-*R*^2^ value of 0.9905 and variation value of 0.72%, which show that the model is accurate and reliable.

### 3.4. Adequacy of the Regression Model

**Figure 4:**
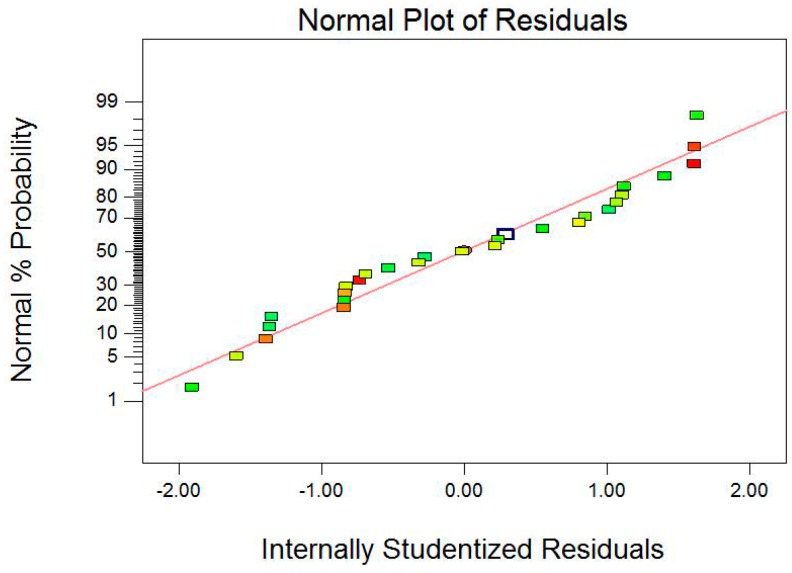
Normal plot of studentized residuals verses normal percent probability.

**Figure 5:**
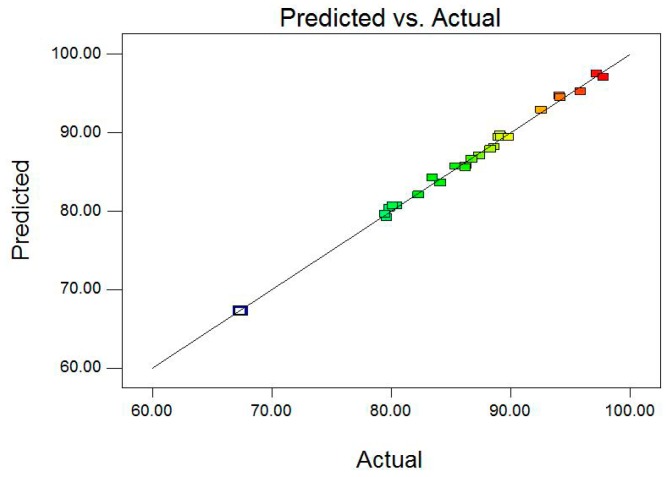
The relationship of predicted and actual value for the BBD.

It is necessary to verify the fitted model to ensure that adequate approximation to the actual values is achieved. The model without adequate analysis may give disingenuous results [43]. Studentized residuals against predicted probability values can judge the model accuracy, and a small residual value shows that prediction of the model is highly accurate. The plots of studentized residuals against predicted probability values show the homogeneously distributed data about either side of line indicating the suitability of the model (Figure 4). Figure 5 shows the relationship between the actual and predicted values for the removal efficiency of Pb(II) ions. It is seen that the experimental results are in good agreement with the predicted value (Figure 5), which implicates that this model can be used in this removal process.

### 3.5. Three Dimensional (3D) Response Surfaces

**Figure 6:**
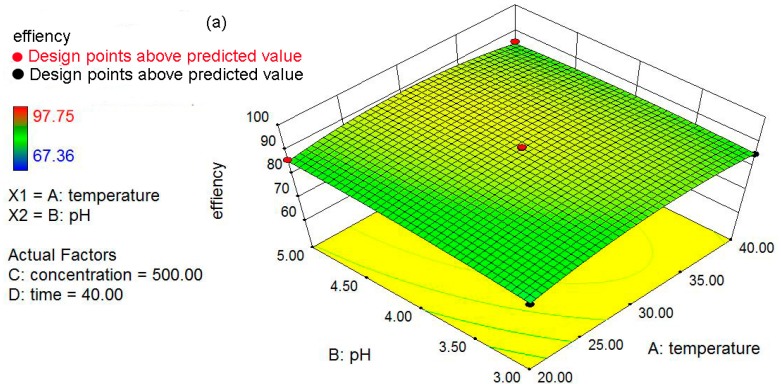

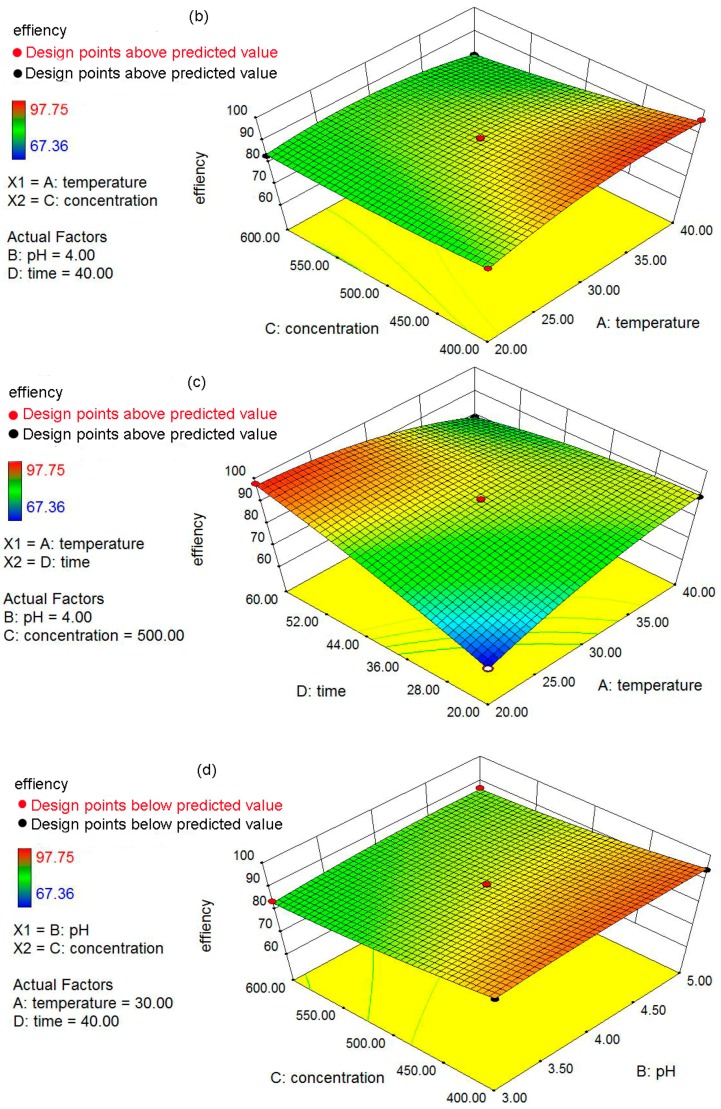

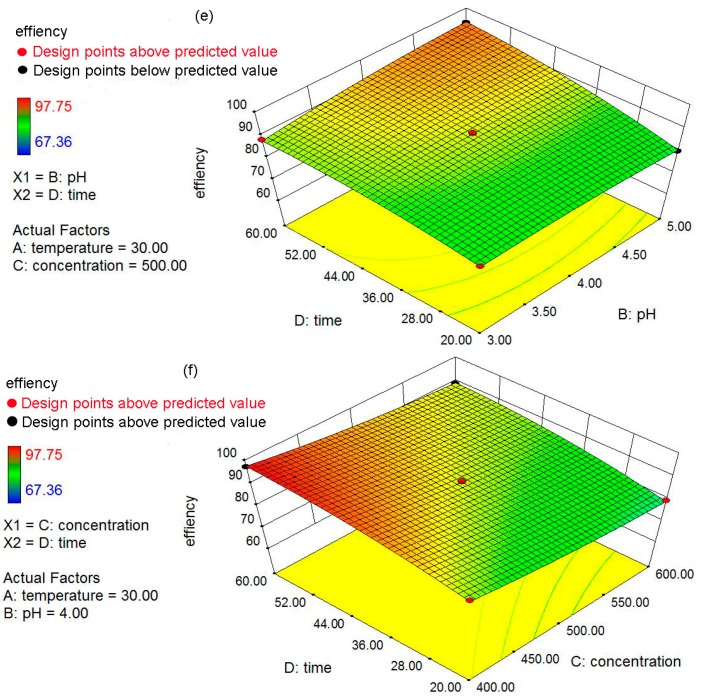
The 3D plots showing effect of temperature and pH (**a**); temperature and initial concentration (**b**); temperature and contact time (**c**); pH and concentration (**d**); pH and contact time (**e**); and concentration and contact time on the removal of Pb(II) ions (**f**).

To better study and understand the effects of the independent variables and their interactions on the response, 3D response surface plots are employed (Figure 6). The response surface plots showed the influence of two variables on the response at the center level of other variables. The nonlinear nature of 3D response surface plots could demonstrate that there are interactions between each of the independent variables and dependent variable. Figure 6a shows the interactive effect between the pH (3–5) and temperature (20–40 °C) on the removal percentage of Pb(II) ions and the response is increased with a rise in temperature and pH. A lower removal percentage was observed under highly acidic conditions, as most of the active sites in nZVI/rGO composites were occupied by H^+^. As pH increases, deprotonation started and the metal ions were complexed with the nZVI/rGO composites, hence the removal percentage of Pb(II) ions rose. Figure 6 also shows the interactive effects between the temperature and initial concentration, the temperature and contact time, the pH and initial concentration, the pH and contact time, and the initial concentration and contact time. 

### 3.6. Optimization for the Removal Percentage of Pb(II) Ions

Optimization of the process variables to maximize the removal percentage of Pb(II) ions by the nZVI/rGO composites from the aqueous solution was performed using the quadratic model within the investigated experimental range of various process variables. The process optimization modeling indicated that the optimal parameters (T = 21.30 °C, pH = 5.00, C = 400.00 mg/L and t = 60.00 min) gave the 100.37% removal percentage for Pb(II) ions, which is satisfactory though with a certain amount of error. The three confirmation experiments were conducted at selected optimal levels of the process parameters. The relative error between average removal percentage obtained through confirmation experiments and the predicted value of the model was within 5%. It should be noted that the optimal value was valid within the specified range of process parameters, and any extrapolation and interpolation should be confirmed through additional experiments.

### 3.7. Adsorption Isotherm

**Figure 7:**
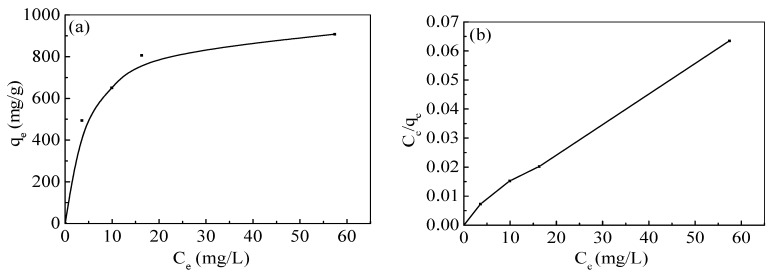
Adsorption isotherm of Pb(II) ions by the nZVI/rGO composites (**a**); and the corresponding linear variation of *C_e_*/*q_e_* with *C_e_* of the nZVI/rGO composites (**b**) (pH = 5.0; nZVI/rGO composites dosage = 0.03 g; temperature = 20 °C; and contact time = 1 h).

The adsorption isotherm of Pb(II) ions by nZVI/rGO composites is shown in Figure 7a. The amount of Pb(II) ions adsorbed on the nZVI/rGO increased with an increase in initial Pb(II) concentration, because higher initial concentrations provided a higher driving force for the ion transportation from the solution to the nZVI/rGO composites, resulting in an increase in collisions between Pb(II) ions and active site on the nZVI/rGO composites. The relationship between C_e_/q_e_ and C_e_ indicated that the adsorption data of Pb(II) ions by the nZVI/rGO composites were fitted well to the Langmuir isotherm (Figure 7b). As shown in Table 4, the correlation coefficient of the Langmuir isotherm was higher than that of the Freundlich isotherm, which indicated that the adsorption of Pb(II) ions by the nZVI/rGO composites was monolayer physical adsorption. The fact that the experimental data were fitted to the Langmuir isotherm may be due to the homogeneous distribution of active sites on the surface of nZVI/rGO composites, since the Langmuir equation assumes that the surface is homogeneous. The Freundlich isotherm assumes that the uptake of metal ions occurs on a heterogeneous surface by multilayer adsorption and that the amount of metal ions adsorbed increases infinitely with an increase in the concentration of ions. Adsorption data fitted to the Langmuir isotherm model with a correlation coefficient of 0.9952 also showed that the removal of Pb(II) ions was governed by the adsorption of nZVI/rGO. The essential characteristics of the Langmuir isotherm can be expressed in terms of a dimensionless constant separation factor *R*_L_, which is defined as:

*R*_L_ = 1/(1 + *kC*_0_) (5)

where *C*_0_ is the initial Pb(II) concentration (mg/L) and k is the Langmuir constant which indicates the nature of adsorption. The value of *R*_L_ (separation factor) indicates the isotherm shape and whether the adsorption is favorable or not [10]. If *R*_L_ > 1, adsorption is unfavorable; *R*_L_ = 1, adsorption is linear; 0 < *R*_L_ < 1, adsorption is favorable; *R*_L_ = 0, adsorption is irreversible. The calculated *R*_L_ values for the adsorption on the nZVI/rGO composites at different initial concentrations of Pb(II) was found in the range from 0.0039 to 0.0078 (Table 5) at 20 °C, showing that the adsorption of nZVI/rGO composites is favorable at the studied temperature.

The experimental value of maximum removal capacity (*C*_max_) of Pb(II) ions by the nZVI/rGO composites was 904 mg/g, which was compared with other materials (Table 6). The *C*_max_ value of Pb(II) ions by the nZVI/rGO composites was higher than that of various materials reported in the previous studies suggesting the great application potential of nZVI/rGO composites for Pb(II) ions removal [9,26,44,45,46,47,48,49,50,51]. Based on the Langmuir isotherm, the *C*_max_ of Pb(II) ions by the nZVI/rGO composites was calculated to be 910 mg/g, which was close to the experimental value.

### 3.8. Removal Kinetics of Pb(II) Ions by nZVI/rGO

**Figure 8:**
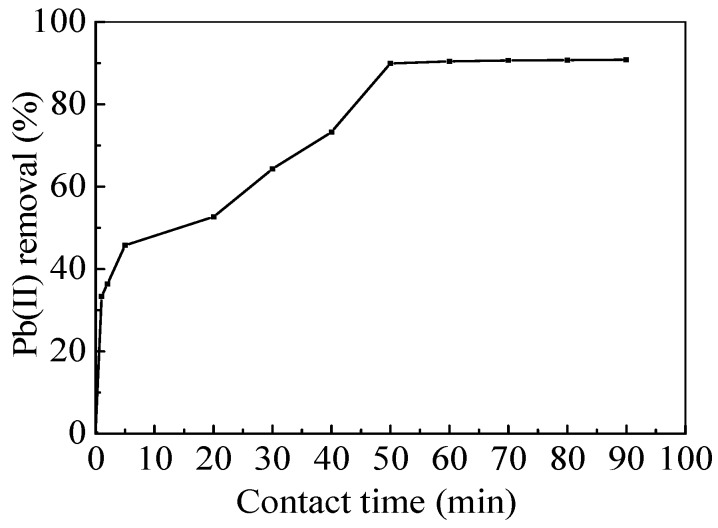
Time dependent study of Pb(II) ions removal by nZVI/rGO composites (pH = 5.0; nZVI/rGO composites dosage = 30 mg; temperature = 20 °C; and Pb(II) concentration = 600 mg/L).

The nZVI/rGO composites were dispersed into Pb(II) solution, which was stirred for various times. After the removal experiments, the nZVI/rGO composites can be easily separated from solution by using a magnet, thus this would provide a convenient and economic way for the separation of nZVI/rGO composites from aqueous solution. For the design of the industrial removal process, it is important to predict the removal kinetic rates. In order to study the removal kinetics of Pb(II) by the nZVI/rGO composites, different kinetic models, such as the pseudo first order model and the pseudo second order model, were used to fit the adsorption kinetic data. The removal kinetics of Pb(II) ions was investigated to determine the required time to achieve equilibrium removal of Pb(II) ions on the nZVI/rGO composites. Figure 8 shows that the full removal process achieved equilibrium within 50 min for the nZVI/rGO composites, and 89.93% of Pb(II) ions was removed by the nZVI/rGO composites with the removal capacity of 899 mg/g. The experimental kinetic data of nZVI/rGO composites were fitted to the pseudo first order kinetic model and pseudo second order kinetic model. Table 7 summarizes the properties of each model. The correlation coefficient of the pseudo first order kinetic model is small compared to those obtained using the pseudo second order model, and as such the pseudo second order model was suitable for describing the removal kinetics. Additionally, the pseudo first order kinetic model showed a large difference in the equilibrium removal capacity (*q_e_*) between the experimental and calculated values, which provided a further evidence for the use of the pseudo second order kinetic model.

### 3.9. Pb(II) Ions Removal Mechanisms

**Figure 9:**
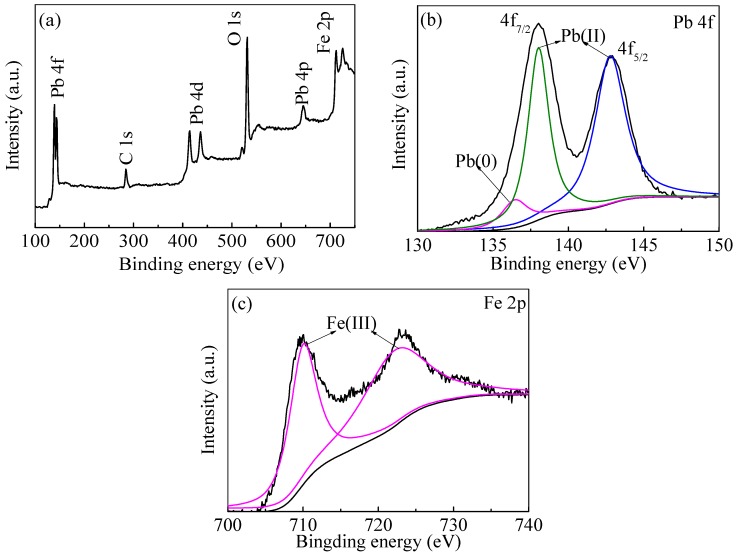
XPS analysis of Pb(II)-reacted nZVI/rGO composites: wide scan of nZVI/rGO composites (**a**); lead 4f of nZVI/rGO composites (**b**); and XPS spectroscopy of Fe 2p in lab made nZVI/rGO composites (**c**) (6000 mg/L Pb(II) solution, pH = 5.0, 300 mg nZVI/rGO composites and time = 1 h).

It was reported in the previous studies that the removal of heavy metal ions from aqueous solution by the nZVI/rGO composites is governed by various mechanisms, including adsorption, complex formation, reduction and precipitation [52,53]. In this study, to investigate the removal mechanisms for the removal of Pb(II) ions by the nZVI/rGO composites, the XPS spectra of both wide scan and high-resolution spectra for Pb 4f of Pb(II)-reacted nZVI/rGO composites are shown in Figure 9. The full survey showed the presence of oxygen, carbon, iron and lead (Figure 9a), which indicated that Pb(II) ions were adsorbed on the surface of nZVI/rGO composites. Pb(0) ions reduced by nZVI were easily immobilized on rGO sheets as has been reported [17,18]. In the high-resolution spectrum of Pb 4f (Figure 9b), a small peak at 136.40 eV (Pb 4f_7/2_) showed that reduction of Pb(II) to Pb(0) also occurred on surface of the nZVI/rGO composites. The small peak also revealed that the removal of Pb(II) ions was controlled by the adsorption of nZVI/rGO, which was probably because this removal process is aerobic. The peaks at 706.99 and 720.60 eV (Figure 9c) disappeared in comparison with Figure 3b, which indicated that the surface of Fe^0^ and Fe^2+^ were changed to Fe^3+^ after reacting with Pb^2+^ ions. The nZVI should be covered by the thin passivation layer (Fe(OH)_3_ and FeOOH) due to the following three aspects [54]. First, the passivation layer is formed in the synthesis of nZVI. Second, the different storage conditions (O_2_ and H_2_O) may facilitate the transformation from Fe(OH)_3_ to FeOOH [54]. With regard to Pb(II) adsorption, Fe(OH)_3_ has two more binding sites for Pb(II) than FeOOH [54]. In fact, the Pb(II) removal capacity of nZVI/rGO composites in this study is significantly higher than in other studies [26,55]. Hence, there is a substantial improvement in the Pb(II) removal capacity of nZVI/rGO with Fe(OH)_3_ shell. This also means that the Fe(OH)_3_ content of shell in this study was higher than in other studies [20,55]. Thirdly, the presence of dissolved oxygen could lead to form a passivation layer on the surface of nZVI in the process for the removal of Pb(II) ions, which suppressed the reduction of Pb(II).

**Figure 10:**
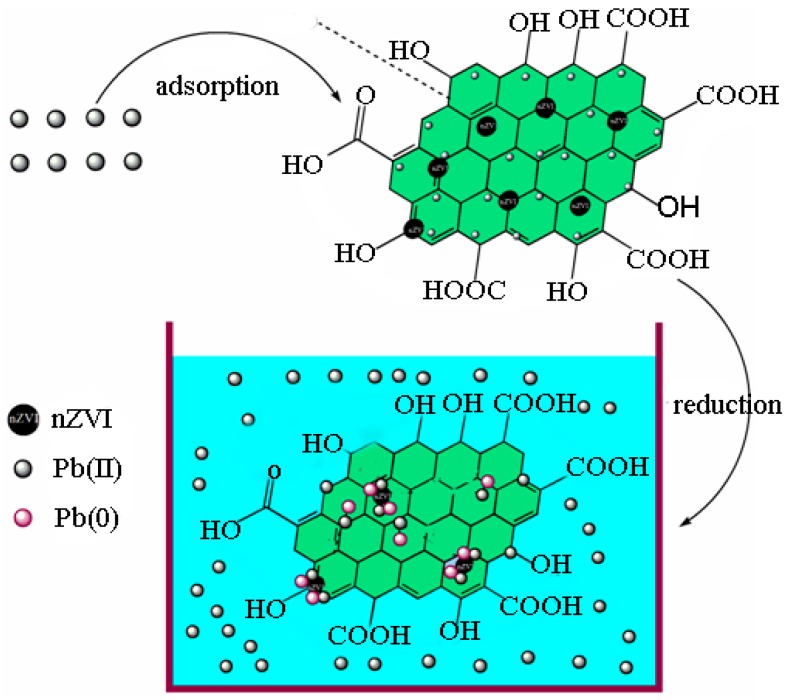
Reduction and adsorption mechanisms for the removal of Pb(II) ions by nZVI/rGO composites.

In addition, another reason for the high removal capacity of Pb(II) ions by the nZVI/rGO composites is that the free Pb(II) ions can be adsorbed on the external surface of rGO sheets through π electron and cation interactions [56]. The process for Pb(II) ions removal should occur in the following steps: free Pb(II) ions were absorbed on the nZVI/rGO composites, and only a small part of Pb(II) ions was reduced to Pb(0) by nZVI (Figure 10). This caused the nZVI/rGO composites containing both metallic and bivalent forms of Pb. Therefore, the adsorption of Pb(II) ions plays a major role in the removal process.

## 4. Conclusions

In the present study, the nZVI/rGO composites were prepared by chemical deposition method, which were characterized by SEM, XRD, Raman spectroscopy, N_2_-sorption and XPS. Using RSM with the quadratic model, the optimal temperature, pH, initial concentration and contact time for Pb(II) ions removal by the nZVI/rGO composites in the present experiment were 21.30 °C, 5.00, 400.00 mg/L and 60.00 min, respectively. The adsorption results could be fitted to the Langmuir isotherm, and the removal kinetics of Pb(II) ions was well described by the pseudo-second order kinetic model. This study suggests that the removal of Pb(II) ions by the nZVI/rGO composites was a physical adsorption. The maximum removal capacity of Pb(II) ions by the nZVI/rGO composites was 904 mg/g, which was higher than that by other materials. In addition, the dominant mechanism for the removal of Pb(II) ions is based on the adsorption by the nZVI/rGO composites. Although the rGO is less cost-effective than other traditional materials, rGO has been synthesized in large scale and at low price in recent years. Therefore, the nZVI/rGO composites have a great potential to be used as suitable material for the immobilization and remediation of heavy metal ions from large volumes of aqueous solutions in environmental cleanup. As prospective material for permeable reactive barriers, the nZVI/rGO composites present a great potential for the removal of Pb(II) ions. Another potential use of these composites is for high-end applications, e.g., advanced treatment of drinking water in some special cases.

## Figures and Tables

**Table 1:** Levels of variables chosen for Box–Behnken design.

Variable	Unit	Factors	Level		
			Low (−1)	Middle (0)	High (+1)
temperature	°C	*X*_1_	20	30	40
pH		*X*_2_	3	4	5
initial concentration	mg/L	*X*_3_	400	500	600
contact time	min	*X*_4_	20	40	60

**Table 2:** Actual and predicted values of Pb(II) ions removal (%).

Run	*X*_1_ °C	*X*_2_	*X*_3_ mg/L	*X*_4_ min	*Y*_Actual_ %	*Y*_Predicted_ %
1	20	4	400	40	86.74 ± 0.19	86.64
2	30	3	600	40	84.13 ± 0.29	83.56
3	20	4	500	60	97.75 ± 0.81	97.10
4	40	5	500	40	86.20 ± 0.44	85.54
5	30	4	500	40	88.99 ± 0.26	89.38
6	40	4	500	60	79.47 ± 0.16	79.58
7	20	4	500	20	67.36 ± 0.22	67.24
8	20	3	500	40	79.87 ± 0.77	80.42
9	30	4	600	20	79.60 ± 0.44	79.19
10	40	4	600	40	80.46 ± 1.12	80.68
11	30	4	500	40	89.50 ± 0.33	89.38
12	40	3	500	40	85.36 ± 0.10	85.70
13	30	4	400	20	88.63 ± 0.62	88.18
14	30	5	500	20	80.12 ± 0.25	80.67
15	30	3	500	20	82.27 ± 0.20	82.05
16	30	5	400	40	94.09 ± 0.19	94.56
17	30	4	500	40	89.83 ± 0.37	89.38
18	30	4	600	60	89.20 ± 0.15	89.54
19	30	3	500	60	88.32 ± 0.88	87.89
20	30	5	500	60	94.15 ± 0.31	94.49
21	30	3	400	40	92.57 ± 0.26	92.91
22	30	4	500	40	89.20 ± 0.28	89.38
23	30	5	600	40	87.39 ± 0.52	87.05
24	40	4	500	20	89.13 ± 0.48	89.78
25	20	4	600	40	83.46 ± 0.41	84.23
26	30	4	400	60	97.20 ± 0.13	97.50
27	20	5	500	40	86.26 ± 0.02	85.81
28	30	4	500	40	89.37 ± 0.32	89.38
29	40	4	400	40	95.87 ± 0.19	95.22

**Table 3:** ANOVA of the second-order polynomial equation.

Source	Sum of Squares	df	Mean Square	*F*-Value	*p*-Value	Remarks
Model	1153.005	14	82.357	209.446	<0.0001	significant
*X*_1_	18.875	1	18.875	48.002	<0.0001	
*X*_2_	20.515	1	20.515	52.172	<0.0001	
*X*_3_	215.562	1	215.562	548.202	<0.0001	
*X*_4_	289.887	1	289.887	737.220	<0.0001	
*X*_1_*X*_2_	7.701	1	7.701	19.584	0.0006	
*X*_1_*X*_3_	36.784	1	36.784	93.547	<0.0001	
*X*_1_*X*_4_	401.001	1	401.001	1019.798	<0.0001	
*X*_2_*X*_3_	0.757	1	0.757	1.925	0.1870	
*X*_2_*X*_4_	15.920	1	15.920	40.487	<0.0001	
*X*_3_*X*_4_	0.265	1	0.265	0.675	0.4253	
*X*_1_^2^	100.238	1	100.238	254.920	<0.0001	
*X*_2_^2^	7.581	1	7.581	19.280	0.0006	
*X*_3_^2^	10.057	1	10.057	25.576	0.0002	
*X*_4_^2^	26.528	1	26.528	67.466	<0.0001	
Residual	5.505	14	0.393			
Lack of Fit	5.104	10	0.510	5.085	0.0655	not significant
Pure Error	0.401	4	0.100			
Total	1158.510	28				

**Table 4:** Langmuir and Freudlich isotherm parameters for the removal of Pb(II) ions by nZVI/rGO composites.

Isotherms	Equation	Parameters	Value of Parameters
Langmuir	$\frac{C_{e}}{q_{e}}=\frac{1}{q_{m}k}+\frac{C_{e}}{q_{m}}$	*k* (L/mg)	0.423
		*q_m_* (mg/g)	910
		*R*^2^	0.9952
Freundlich	$\log q_{e}=\log K_{f}+\frac{1}{n}\log C_{e}$	*K_f_* (mg/g)	391.03
		*n*	4.52
		*R*^2^	0.9203

**Table 5:** Data showing the *R*_L_ values obtained for the adsorption of Pb(II) ions by nZVI/rGO.

Initial Concentration (mg/L)	*R*_L_ Value
300	0.0078
400	0.0059
500	0.0047
600	0.0039

**Table 6:** Removal capacities of Pb(II) ions by nZVI/rGO composites and other materials.

Materials	*C*_max_ (mg/g)	Reference
6% nZVI/rGO composites	556	[26]
nZVI	364	[26]
Tephrosia Purpuria Leaf	100	[9]
Bael leaves (Aegle marmelos)	104	[44]
carbon nanotubes	102	[45]
hydroxyaptite/chitosan	264	[46]
Cucumissativus peel	28	[47]
Mn-Fe/MnO_2_	261	[48]
silica/nano zero-valent iron	416	[49]
attapulgite	845	[50]
Ni-doped bamboo charcoal	143	[51]
33.3% nZVI/rGO composites	904	Present study

**Table 7:** Kinetic parameters for the removal of Pb(II) ions by nZVI/rGO composites.

Model	Equation	Parameters	Value of Parameters
First-order kinetics	$\log(q_{e}-q_{t})=\log q_{e}-k_{1}t/2.303$	*k*_1_ (1/min)	3.16 × 10^−2^
		*k*_m_ (1/min·mg)	1.05 × 10^−3^
		*k*_SA_ (1/min·m^2^)	2.69 × 10^−2^
		*q_e_* (mg/g)	652
		*R*^2^	0.8908
Second-order kinetics	$t/q_{t}=1/k_{2}{q^{2}}_{e}+t/q_{e}$	*k*_2_ (g/mg·min)	3.41 × 10^−1^
		*q_e_* (mg/g)	714
		*R*^2^	0.9726

The observed rate constant (*k*_1_) was normalized by the mass of nZVI/rGO (*k*_m_) composites and by the surface area (*k*_SA_) of nZVI/rGO composites.

## Supplementary Materials

The following are available online at www.mdpi.com/1996-1944/9/8/687/s1. Figure S1: Effect of temperature on Pb(II) removal by nZVI/rGO composites: pH = 5.0; nZVI/rGO composites dosage = 30 mg; Pb(II) concentration = 600 mg/L; and time = 1 h (a). Effect of pH on Pb(II) removal by nZVI/rGO composites: Temperature = 20 °C; nZVI/rGO composites dose = 30 mg; Pb(II) concentration = 600 mg/L; and time = 1 h (b). Effect of initial Pb(II) concentration removal by nZVI/rGO composites: Temperature = 20 °C; pH = 5.0; nZVI/rGO composites dose = 30 mg; and time = 1 h (c). Effect of contact time on Pb(II) removal by nZVI/rGO composites: Temperature = 20 °C; pH = 5.0; nZVI/rGO composites dose = 30 mg; and Pb(II) concentration = 600 mg/L (d); Figure S2: SEM image of nZVI (a) and the corresponding magnified images (b–d) respectively; Figure S3: SEM image of nZVI/rGO composites (a) and the corresponding magnified images (b–d) respectively.
