# Supplementary material for: Synthesis and Characterization of Reduced Graphene Oxide-Supported Nanoscale Zero-Valent Iron (nZVI/rGO) Composites Used for Pb(II) Removal
Source: Materials (Basel). 2016 Aug 12;9(8):687. doi: 10.3390/ma9080687 (PMC5512353; doi:10.3390/ma9080687)
Supplement: Supplementary file 1 [file materials-09-00687-s001.pdf]

# Supplementary Materials: Synthesis and Characterization of Reduced Graphene Oxide-Supported Nanoscale Zero-Valent Iron (nZVI/rGO) Composites Used for Pb(II) Removal

**Mingyi Fan, Tongjun Li, Jiwei Hu, Rensheng Cao, Qing Wu, Xionghui Wei, Lingyun Li, Xuedan Shi and Wenqian Ruan**

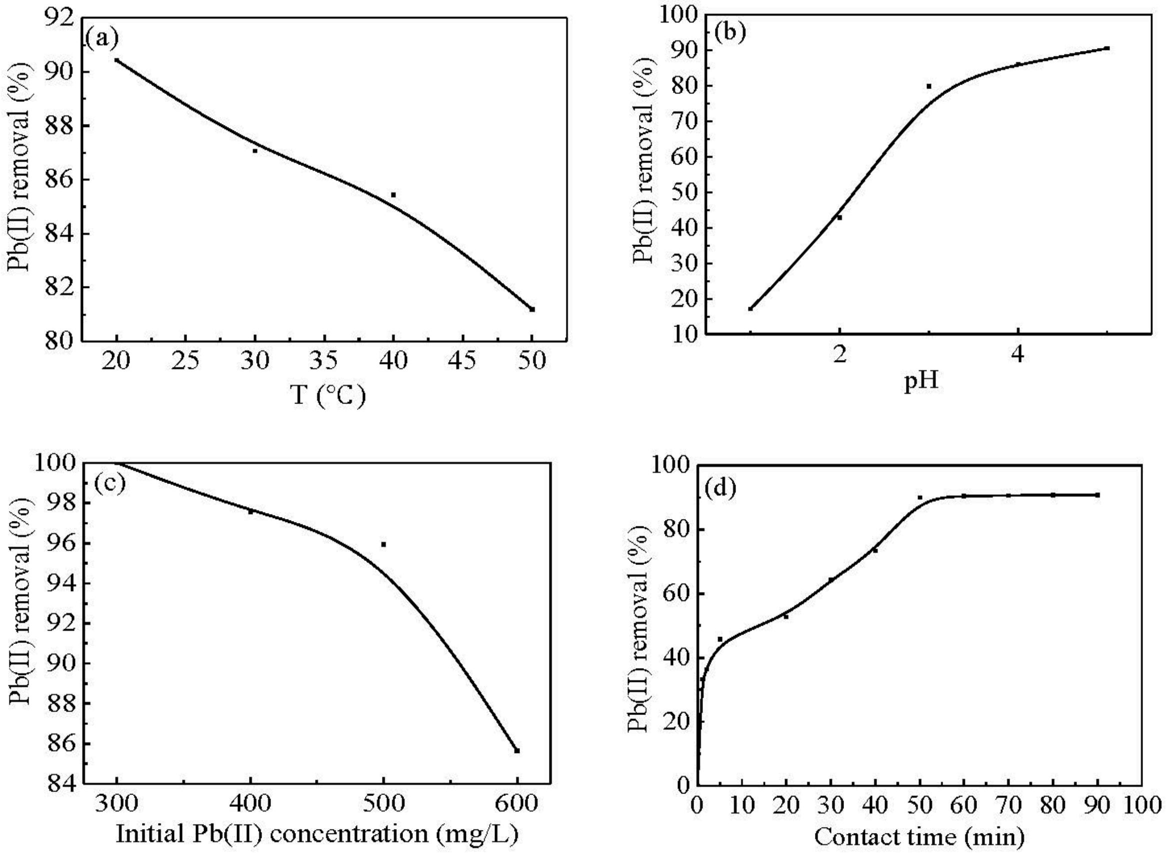


**Figure S1.** Effect of temperature on Pb(II) removal by nZVI/rGO composites: pH = 5.0; nZVI/rGO composites dosage = 30 mg; Pb(II) concentration = 600 mg/L; and time = 1 h (**a**). Effect of pH on Pb(II) removal by nZVI/rGO composites: Temperature = 20 °C; nZVI/rGO composites dose = 30 mg; Pb(II) concentration = 600 mg/L; and time = 1 h (**b**). Effect of initial Pb(II) concentration removal by nZVI/rGO composites: Temperature = 20 °C; pH = 5.0; nZVI/rGO composites dose = 30 mg; and time = 1 h (**c**). Effect of contact time on Pb(II) removal by nZVI/rGO composites: Temperature = 20 °C; pH = 5.0; nZVI/rGO composites dose = 30 mg; and Pb(II) concentration = 600 mg/L (**d**).

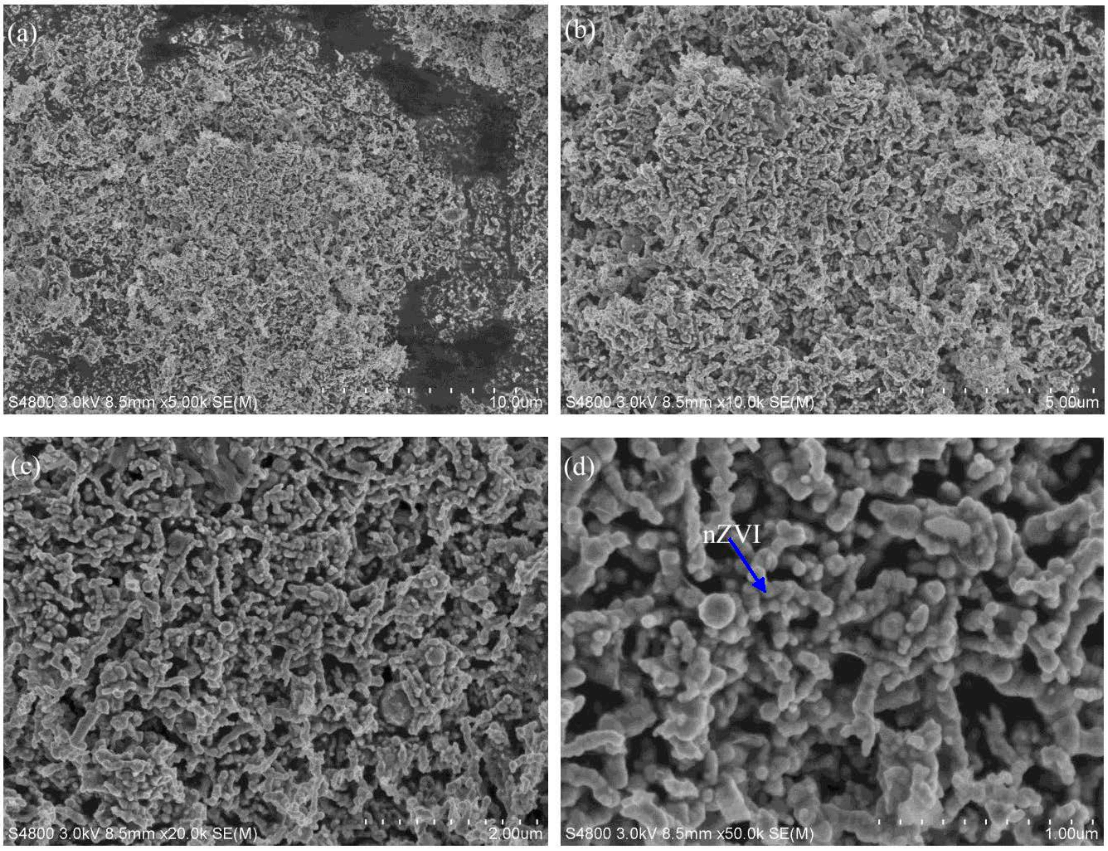


**Figure S2.** SEM image of nZVI (**a**) and the corresponding magnified images (**b**–**d**) respectively.

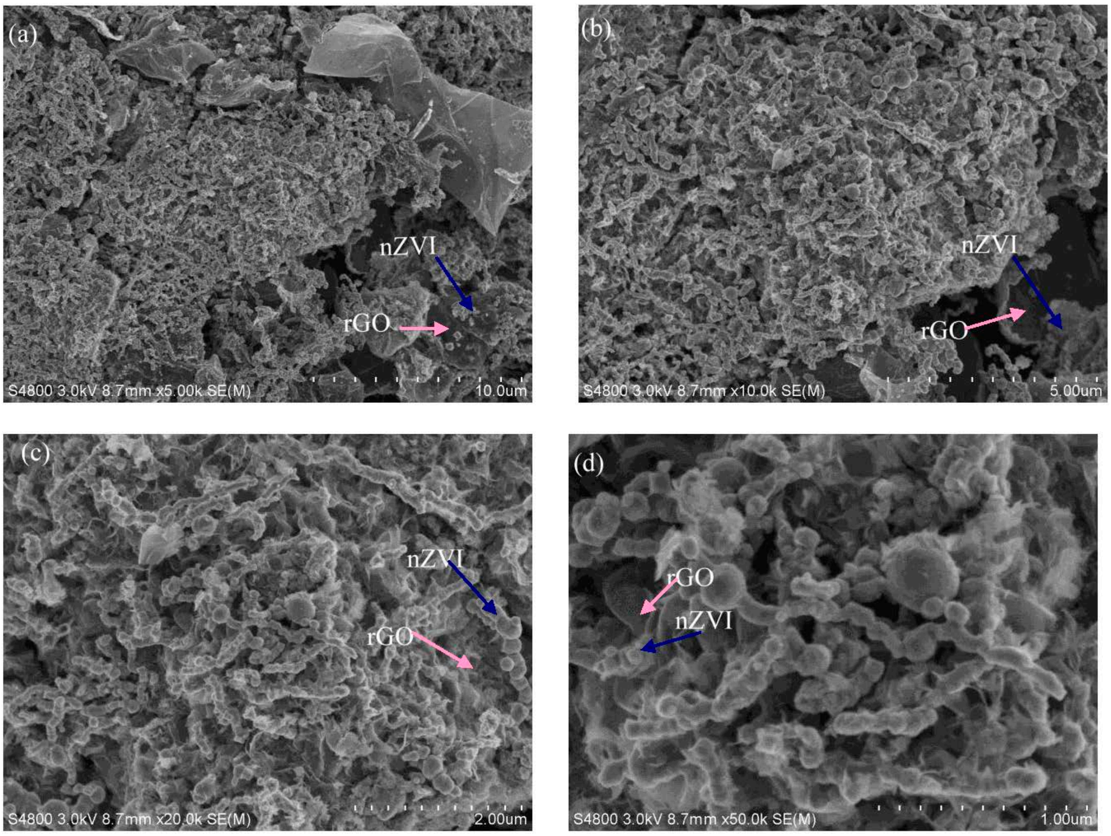


**Figure S3.** SEM image of nZVI/rGO composites (**a**) and the corresponding magnified images (**b**–**d**) respectively.
